# Deaths in Philadelphia from Aug. 24th to Sept. 21st, 1850

**Published:** 1850-10

**Authors:** James Aitken Meigs

**Affiliations:** Student of Medicine


*Deaths in Philadelphia from Aug. 24th to Sept. 21st*, 1850. Reported by Mr. JAMES AITKEN MEIGS, Student of Medicine.

| Diseases. | Ad'ts | Chil. | Diseases. | Ad'ts | Chil. |
|---|---|---|---|---|---|
| Abscess, | 0 | 1 | Fever, typhoid, | 11 | 3 |
| Anæmia, | 0 | 1 | " typhus, | 6 | 0 |
| Angina pectoris, | 1 | 0 | Gangrene, | 1 | 1 |
| Apoplexy, | 6 | 0 | Hæmoptysis, | 2 | 1 |
| Asthma, | 2 | 0 | Hemorrhage | 1 | 1 |
| Burns, | 1 | 2 | " uterine, | 3 | 0 |
| Cachexia, | 0 | 1 | Ileus, | 1 | 0 |
| Cancer, | 2 | 0 | Inflammation of brain, | 5 | 16 |
| " breast, | 1 | 0 | " bronchi, | 0 | 10 |
| " stomach, | 2 | 0 | " liver, | 2 | 1 |
| " uterus, | 2 | 0 | " lungs, | 5 | 14 |
| Cancrum oris, | 0 | 2 | " ovaries, | 1 | 0 |
| Caries of jaw | 0 | 1 | " peritoneum, | 5 | 2 |
| Casualties, | 1 | 1 | " pleura, | 0 | 2 |
| Cholera infantum, | 0 | 48 | " stom. & bowels, | 10 | 11 |
| Compression of brain, | 0 | 1 | " throat, | 0 | 1 |
| Concussion " | 0 | 1 | Inanition, | 0 | 5 |
| Congestion of lungs, | 0 | 2 | Intemperance, | 6 | 0 |
| " brain, | 2 | 6 | Ischuria, | 1 | 0 |
| Convulsions, | 2 | 27 | Jaundice, | 1 | 0 |
| Croup, | 0 | 7 | Kidneys and liver, fatty degeneration of, | 1 | 0 |
| Cyanosis, | 0 | 2 | Malformation, | 0 | 1 |
| Debility, general, | 5 | 11 | " of heart, | 0 | 1 |
| Diabetes, | 2 | 0 | Malignant disease of bones, | 0 | 1 |
| Diarrhœa, | 8 | 16 | " sore throat, | 0 | 1 |
| Disease of brain, | 0 | 8 | Mania-a-potu, | 3 | 0 |
| " heart, | 5 | 1 | Marasmus, | 0 | 34 |
| " kidneys, | 1 | 1 | Measles, | 0 | 2 |
| " liver, | 2 | 0 | Mortification of hand, | 1 | 0 |
| " lungs, | 2 | 1 | Obstruction of bowels, | 1 | 0 |
| " ovaries, | 1 | 0 | Old age, | 22 | 0 |
| " stomach and bowels, | 0 | 2 | Ossificat. of valves of heart, | 1 | 0 |
| Dropsy, | 6 | 4 | Palsy, | 6 | 0 |
| " abdominal, | 1 | 0 | Pertussis, | 0 | 21 |
| " of breast, | 1 | 0 | Phthisis pulmonalis, | 47 | 13 |
| " head, | 0 | 26 | Poisoning, | 2 | 0 |
| Drowned, | 7 | 7 | Purpura hemorrhagica, | 0 | 2 |
| Dysentery, | 33 | 41 | Rupture of uterus | 1 | 0 |
| Effusion on brain, | 2 | 7 | Scrofula, | 1 | 2 |
| Epilepsy, | 2 | 0 | Small pox, | 0 | 2 |
| Erysipelas, | 1 | 0 | Softening of brain, | 0 | 1 |
| Fever, | 1 | 3 | Spina bifida, | 0 | 2 |
| " cerebral, | 0 | 1 | Still born, | 0 | 40 |
| " congestive, | 1 | 0 | Suffocation, | 2 | 1 |
| " hectic, | 0 | 1 | Syncope, | 1 | 0 |
| " intermittent, | 1 | 3 | Syphilis, | 1 | 0 |
| " puerperal, | 1 | 0 | Tabes mesenterica, | 1 | 5 |
| " remittent, | 6 | 2 | Teething, | 0 | 8 |
| " scarlet, | 0 | 12 | | | |

| Diseases. | Ad'ts | Chil. | Diseases. | Ad'ts | Chil. |
|---|---|---|---|---|---|
| Tetanus, . . . . | 0 | 1 | Unknown, . . . . | 7 | 2 |
| Ulceration of small intest. | 2 | 1 | Violence, . . . . | 4 | 0 |
| " colon, . . | 1 | 0 | Wound, . . . . | 2 | 0 |
| | | | | 269 | 459 |

Total, . . . . . . . . . . . . . . 728

Of the foregoing the ages were as follows:—

| | | | | |
|---|---|---|---|---|
| Under | 1 year, | | | 207 |
| From | 1 | to | 2, | 116 |
| | 2 | - | 5, | 82 |
| | 5 | - | 10, | 26 |
| | 10 | - | 15, | 13 |
| | 15 | - | 20, | 15 |
| | 20 | - | 30, | 64 |
| | 30 | - | 40, | 61 |
| | 40 | - | 50, | 40 |
| | 50 | - | 60, | 32 |
| | 60 | - | 70, | 31 |
| | 70 | - | 80, | 21 |
| | 80 | - | 90, | 17 |
| | 90 | - | 100, | 3 |
| | | | | 728 |

Included in this number, are 53 from the Almshouse, 14 from the surrounding country, and 58 people of color.

